# Plurihormonal Cosecretion by a Case of Adrenocortical Oncocytic Neoplasm

**DOI:** 10.1155/2016/6785925

**Published:** 2016-06-16

**Authors:** J. J. Corrales, C. Robles-Lázaro, A. I. Sánchez-Marcos, M. C. González-Sánchez, P. Antúnez-Plaza, J. M. Miralles

**Affiliations:** ^1^Servicio de Endocrinología y Nutrición, Hospital Clínico Universitario, Paseo de San Vicente No. 58, 37007 Salamanca, Spain; ^2^Departamento de Medicina, Universidad de Salamanca, Campus Miguel de Unamuno s/n, 37007 Salamanca, Spain; ^3^Centro de Investigación del Cáncer (IBMCC-CSIC/USAL) and Instituto Biosanitario de Salamanca, Campus Miguel de Unamuno s/n, 37007 Salamanca, Spain; ^4^Unidad de Cirugía Endocrina, Departamento de Cirugía, Hospital Clínico Universitario de Salamanca, Paseo de San Vicente No. 58, 37007 Salamanca, Spain; ^5^Servicio de Anatomía Patológica, Departamento de Anatomía Patológica, Hospital Universitario de Salamanca, Paseo de San Vicente No. 58, 37007 Salamanca, Spain

## Abstract

Adrenocortical oncocytic neoplasms (oncocytomas) are extremely rare; only approximately 159 cases have been described so far. The majority are nonfunctional and benign. We describe an unusual case of a functional oncocytoma secreting an excess of glucocorticoids (cortisol) and androgens (androstenedione and DHEAS), a pattern of plurihormonal cosecretion previously not reported in men, presenting with endocrine manifestations of Cushing's syndrome. The neoplasm was considered to be of uncertain malignant potential (borderline) according to the Lin-Weiss-Bisceglia criteria.

## 1. Background

Adrenocortical oncocytic neoplasms (oncocytomas) are very rare tumours. Since the first description by Kakimoto et al. [1] in 1986, only 159 cases have been reported [2], and they are usually nonfunctional and benign [3]. Only 17% of the adrenal oncocytomas are functional [3]. Herein we report a rare case of a functional adrenocortical oncocytoma cosecreting cortisol, androstenedione, and DHEAS.

## 2. Case Presentation

A 58-year-old man was referred in May 2013 with lumbar persistent pain on the left side. A recent diagnosis of hypertension and type 2 diabetes was established. The examination revealed some features leading to suspicion of Cushing's syndrome, such as central obesity, round face, fine skin, and capillary fragility.

## 3. Investigation

The endocrine study confirmed the suspicion of Cushing syndrome and showed cosecretion of increased amounts of DHEAS and androstenedione (Table 1). The measurements of urinary metanephrines, epinephrine, norepinephrine, and dopamine were normal.

An abdominal computed tomography (CT) scan revealed a 9.3 × 7 × 10 cm left adrenal mass. The radiological description was a heterogeneous mass, with intratumoral calcifications, hypodense areas suggesting necrosis, and enhanced imaging after contrast administration. The neoplasm was suspected to correspond to an adrenal carcinoma. Bone densitometry revealed osteoporosis with a *T* score of −3.2 SD in the vertebral bone and −1.9 SD in the femur. A bone scan revealed pathological deposits in several vertebral bodies with vertebral crushing.

## 4. Treatment

A left adrenalectomy was performed. Macroscopically, the mass was adhered to the spleen and the tail of the pancreas and, therefore, considering the clinical and radiological suspicion of malignancy, apart from the adrenalectomy, both the spleen and the tail of the pancreas were excised. The postoperative course was uneventful.

**Figure 1:**
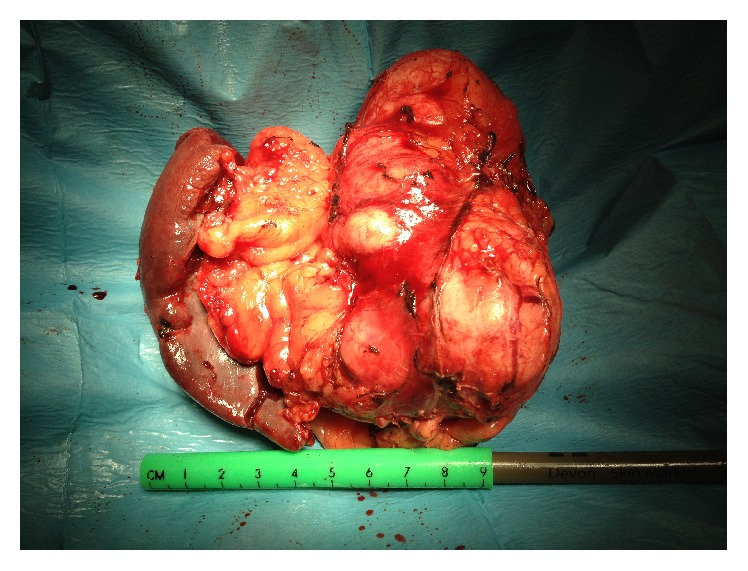
Macroscopic image of the oncocytoma.

**Figure 2:**
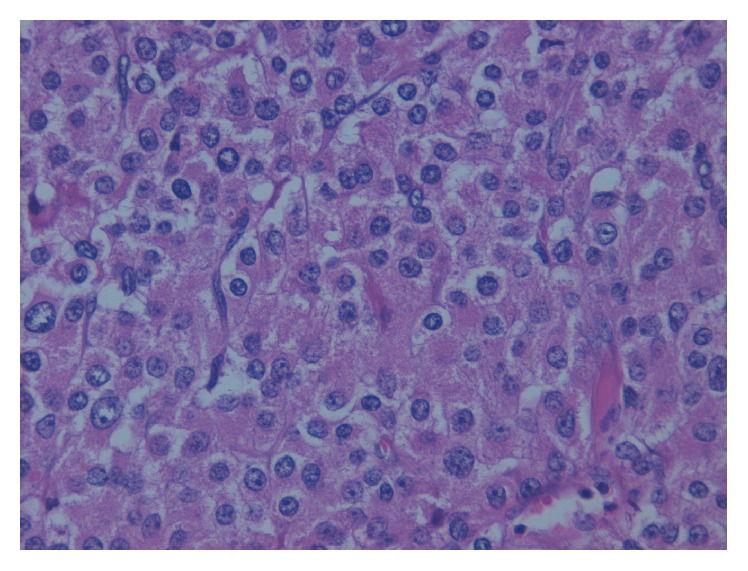
Microscopic features of the adrenocortical neoplasm with abundant eosinophilic and granular cytoplasm (H.E. ×20).

At the pathologic examination, macroscopically the tumour measured 15 × 10 × 8 cm and weighed 361.98 g (Figure 1). The microscopic appearance of multiple tissue samples shows typical oncocytic cells, which are characterized by abundant eosinophilic and granular cytoplasm due to the accumulation of mitochondria (Figure 2).

There were both capsular and sinusoidal invasion with extended necrosis and calcifications. Less than 5 mitoses per HPF were present. Neither atypical mitoses nor venous invasion was detected. Immunohistochemistry showed the tumour cells to be positive for synaptophysin, chromogranin A, inhibin-*α* + in 25% of tumoral cells, and MIB-1 in 10%. The cells were negative for melan A and calretinin.

## 5. Outcome and Follow-Up

After the surgical intervention, the patient showed data of postoperative adrenal insufficiency that required corticoid replacement therapy. There was an improvement of the clinical features of Cushing's syndrome with normalization of the cortisol and the adrenal androgen plasma levels. The values of basal cortisol in plasma fell to 114 nmol/L, those of androstenedione to less than 1 nmol/L, and those of DHEAS to 1.1 *µ*mol/L. A control of bone densitometry, 15 months after surgery, revealed an improvement of 17.2% at vertebral bone without changes in the femur. The radiological follow-up of the patient included thoraco-abdomino-pelvic computed tomography scan that was performed every 3 months, as well as ^18^FDG-PET/CT showing only images of what remains of the surgical scars, without evidence of recurrence of Cushing's syndrome, the tumour, or metastasis 30 months after the surgical treatment.

## 6. Discussion

This patient presented with an exceptional adrenocortical oncocytoma of uncertain malignant potential (borderline), according to the Lin-Weiss-Bisceglia criteria for the specific assessment of the malignant potential of oncocytic adrenocortical neoplasms [4], cosecreting an excess of cortisol, DHEAS, and androstenedione. Our patient does not have any of the 3 major criteria needed to be classified as malignant oncocytic neoplasm, according to the Lim-Weiss-Bisceglia criteria [4], such as high mitotic rate (>5 per 50 HPF), atypical mitotic figures, and venous invasion. However, the patient meets all the four minor criteria used to define oncocytic neoplasms of uncertain malignant potential (borderline), including large size (>10 cm) and/or huge weight (>200 g), extended necrosis, capsular invasion, and sinusoidal invasion. The presence of only one minor criterion allows considering the tumour to have uncertain malignant potential (borderline). Among the functional borderline adrenal oncocytomas described so far, to the best of our knowledge, this is the first to associate such hormonal pattern.

The histological findings do not allow us to ascertain the evolution of these borderline adrenal oncocytomas and the low number of cases so far described precludes us from establishing an informed prognosis. In addition, little information exists about the natural history as well as the outcome of these borderline oncocytomas, creating uncertainties for both the patient and the physician [5]. Therefore the information about clinical characteristics in the few cases reported may help in the management of the patients. Bisceglia et al. [4] did not find recurrence in 4 patients followed from 6 to 10 months after surgical treatment. No relapse was found in another two cases followed by Lin et al. [6] for 12 and 19 months. In a report of Wong et al. [7] showing the best information so far about the prognosis of these rare tumours, local recurrence or distant metastases occurred in approximately 40% for patients with borderline oncocytic neoplasms after 150 months of follow-up after diagnosis and the cumulative survival after this time was 80%. After 30 months of follow-up, our patient was disease-free, without any evidence of clinical, biochemical, or morphological recurrence. Nevertheless, 30 months is a too short period of follow-up to exclude future relapse, according to the above-described finding of Wong et al. [7] about the prognosis of these tumours.

The importance of the recognition of this subclass of oncocytomas derives from their uncertain biologic behaviour and the variable form of presentation in that hormone hypersecretion is not as infrequent as previously believed. Mearini et al. [3], after a systematic literature search using MEDLINE/Cochrane libraries from 1950 until August 2012, detected 147 cases of adrenal oncocytic neoplasms and most of them were nonfunctional masses. This is the first report of the rare association of an excess of cortisol, DHEAS, and androstenedione cosecretion with Cushing's syndrome by an adrenocortical oncocytoma in men. Previously, such association was described by Logasundaram et al. [8] in a woman presenting with virilism.

In a recent clinicopathologic study of 13 cases by Wong et al. [7] it was pointed out that all but one of oncocytic adrenocortical neoplasms showing endocrine activity were classified as malignant. Our case indicates that the endocrine activity is not always associated with malignancy since it can be found in borderline tumours.

## Figures and Tables

**Table 1:** Hormone measurements in plasma before surgery.

Hormone	Value	Normal range
Cortisol (nmol/L)	764	193–359
ACTH (pmol/L)	<0.4	<15
Androstenedione (nmol/L)	34	1.7–12
DHEAS (*µ*mol/L)	23	0.3–13
Urine cortisol (nmol/L)	798	<413
Cortisol post-1 mg DXM (nmol/L)	772	<50
Testosterone (nmol/L)	10.4	8–34
Estradiol (pmol/L)	88	18–256

## Additional Points

(i) Adrenocortical oncocytomas may cosecrete various steroids. (ii) Clinical, hormonal, and radiologic features may suggest malignant tumours. (iii) Their malignant potential must be assessed using specific pathological criteria.
